# Neuroprotective effects of allicin on ischemia-reperfusion brain injury

**DOI:** 10.18632/oncotarget.22355

**Published:** 2017-11-10

**Authors:** Xiangyi Kong, Shun Gong, Lijuan Su, Chen Li, Yanguo Kong

**Affiliations:** ^1^ Department of Neurosurgery, Peking Union Medical College Hospital, Chinese Academy of Medical Sciences, No. 1 Shuaifuyuan Hutong, Dongcheng District, Beijing, P. R. China; ^2^ Department of Breast Surgical Oncology, National Cancer Center/Cancer Hospital, Chinese Academy of Medical Sciences and Peking Union Medical College, Chaoyang District, Panjiayuan, Nanli 17, Beijing, P. R. China; ^3^ Department of Neurosurgery, Shanghai Institute of Neurosurgery, PLA Institute of Neurosurgery, Shanghai Changzheng Hospital, Second Military Medical University, Shanghai, P.R. China; ^4^ College of Computer Science and Technology, Zhejiang University, Hangzhou, Zhejiang, P.R. China; ^5^ Cancer Epigenetic Laboratory, Department of Clinical Oncology, State Key Laboratory of Oncology in South China, Sir YK Pao Center for Cancer and Li Ka Shing Institute of Health Science, The Chinese University of Hong Kong, Shatin, Hong Kong

**Keywords:** allicin, ischemia-reperfusion brain injury, neuroprotection, oxidative stress

## Abstract

**Background:**

Ischemia-reperfusion brain injury (IRBI) is an important cause for mortality and morbidity. Studies on humans and animals showed that oxidative stress (OS) plays a crucial role in ischemic stroke with or without reperfusion. Allicin is reported to be able to attenuate OS and has neuroprotective effects on rabbits’ ischemia–reperfusion spinal cord injury.

**Aim:**

To explore whether Allicin pretreatment has neuroprotective effects on IRBI in mice.

**Methods and results:**

Transient middle cerebral artery occlusion (MCAO) was conducted to induce IRBI in mice. The mice were pretreated with either Allicin (MCAOA) or normal saline in the same volume (MCAONS). Sham-operated groups [Allicin group (SOA) and normal saline group (SONS)] were also set. Blood pressure and cerebral blood flow measurements revealed comparable hemodynamics. Via brain MRI and neuronal nuclear antigen (NeuN) immune-histochemical staining, MCAOA mice had a significantly reduced stroke size than MCAONS mice (P < 0.05, n = 15). Allicin pretreatment could attenuate the OS, the activity of nicotinamide adenine dinucleotide phosphate (NADPH) oxidase, inflammation, dysfunction of mitochondrial respiratory chain, and apoptosis (all P < 0.05, n = 15). Furthermore, Allicin also increased the activities of endogenous antioxidant enzymes, including catalase (CAT), superoxide dismutase (SOD), glutathione peroxidase (GPX), and glutathione S-transferase (GST), and promoted the angiogenesis in the peri-infarct zone (all P < 0.05, n = 15).

**Conclusion:**

We showed that Allicin could protect mice from IRBI through a series of mechanisms. Allicin represents a new therapeutic direction of IRBI.

## INTRODUCTION

Stroke is one of the most leading global causes of mortality across the world [1]. Despite ongoing advances in stroke imaging and treatment, ischemia-reperfusion brain injury (IRBI) stroke continues to debilitate patients with devastating outcomes at both personal and societal levels. Therefore, it is important to illuminate the exact molecular biological mechanisms underlying the pathogenesis of IRBI and explore more effective therapies.

Oxidative stress (OS) plays important roles in cardia-cerebrovascular diseases [2]. Many human and animal researches demonstrated a correlation between IRBI and increased systemic and local OS [2, 3]. Targeting OS in both primary ischemic-injury and the following reperfusion-injury, lots of medicine, especially natural products extracted from medicinal plants, have been proved to have neuroprotective effects on IRBI [4].

Garlic, as a natural crop, contains rich sulfur-containing amino acids. It has been used as important traditional Chinese medicine with little reported toxicity for centuries in China [5]. Many studies have suggested that the pharmacologic actions of garlic are associated with its biological pharmacological effects like anticancer, antihypertensive, anti-parasitic, anti-fungal, anti-microbial and anti-inflammatory activities [6-9]. In 2012, Zhu, et al.’s research revealed that Allicin could protect against ischemia-reperfusion injury of spinal cord [10]. In 2015, Zhang et al. published a study in Mol Med Rep that explored Allicin’s protective effects against IRBI in rat models [11]. Rats were randomly assigned to the MCAO group, the allicin + MCAO group, and the sham-operation group. Their results demonstrated that Allicin decreased the infarction area, cerebral water content, neuron apoptosis, tumor necrosis factor (TNF-α) expression, and the activities of serum myeloperoxidase (MPO). However, the intervention in their study was administering Allicin after the MCAO procedure (50 mg/kg i.p. after 3 hours of reperfusion daily for 5 consecutive days), which could not be able to evaluate the preventive effects of Allicin on IRBI. Secondly, since the direct effects of Allicin was anti-OS theoretically instead of anti-inflammation, only exploring the serum TNF-α level and MPO activity seems insufficient to elucidate the specific mechanism in Allicin’s protective effects. In this study, we thus more deeply analyzed the preventative and therapeutic effects of Allicin on IRBI. In concrete terms, we explored whether mice that received Allicin preoperatively could be protected from ischemia-reperfusion-induced and OS-mediated brain injury and brain function impairments. Allicin’s effects on mitochondrial signaling pathways which are crucially involved in OS-processes were also investigated.

## RESULTS

### Reduced brain infarction after ischemia-reperfusion injury in MCAOA mice

**Figure 1:**
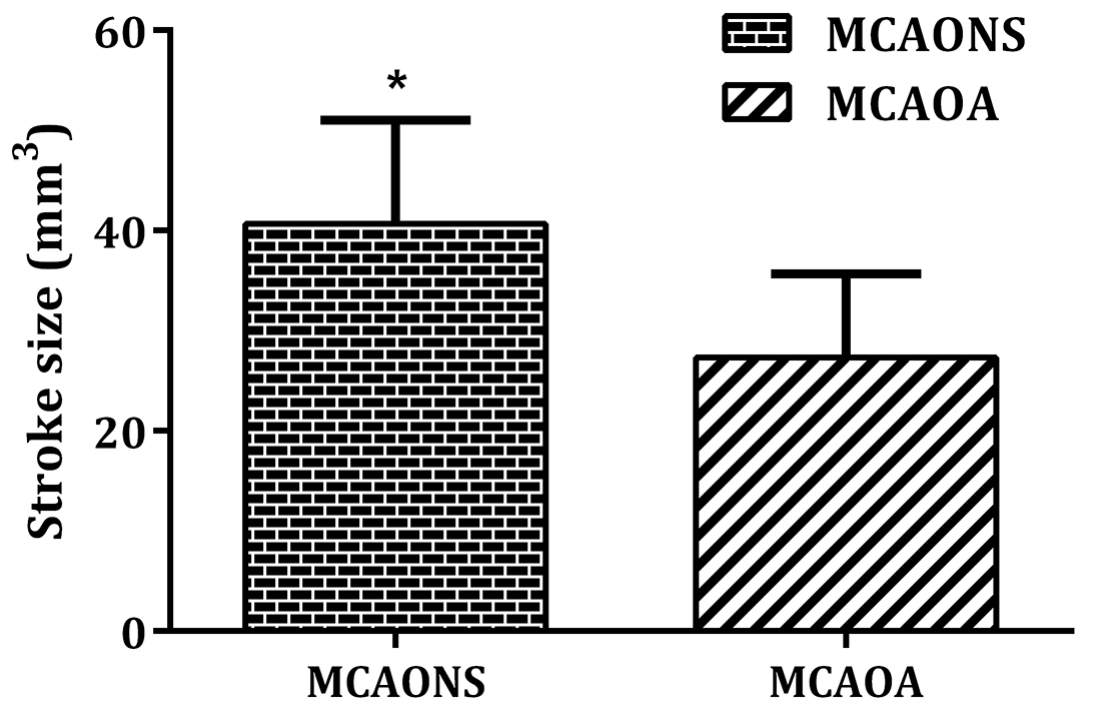
Stroke size measurement after ischemia-reperfusion brain injury through brain MRI (T2WI) 24 h after MCAO in MCAONS mice and MCAOA mice MCAOA mice show reduced stroke size compared with MCAONS mice, ^*^P < 0.05. Values are given as cubic millimeter.

Following 24 h of reperfusion, both MCAOA and MCAONS mice underwent a comparable weight loss (data not shown). To quantify stroke size, mice underwent brain MRI scannings [12]. Morphometrical analysis revealed a significantly reduced stroke size in MCAOA mice compared with MCAONS mice (MCAOA: 27.33 ± 8.33 mm^2^ vs. MCAONS: 40.65 ± 10.35 mm^2^; ^*^p < 0.05; n = 15; Figure 1). SOA and SONS mice did not display any lesions (data not shown).

### Allicin improves neurological function following MCAO

#### Neurological severity score (NSS)

**Figure 2:**
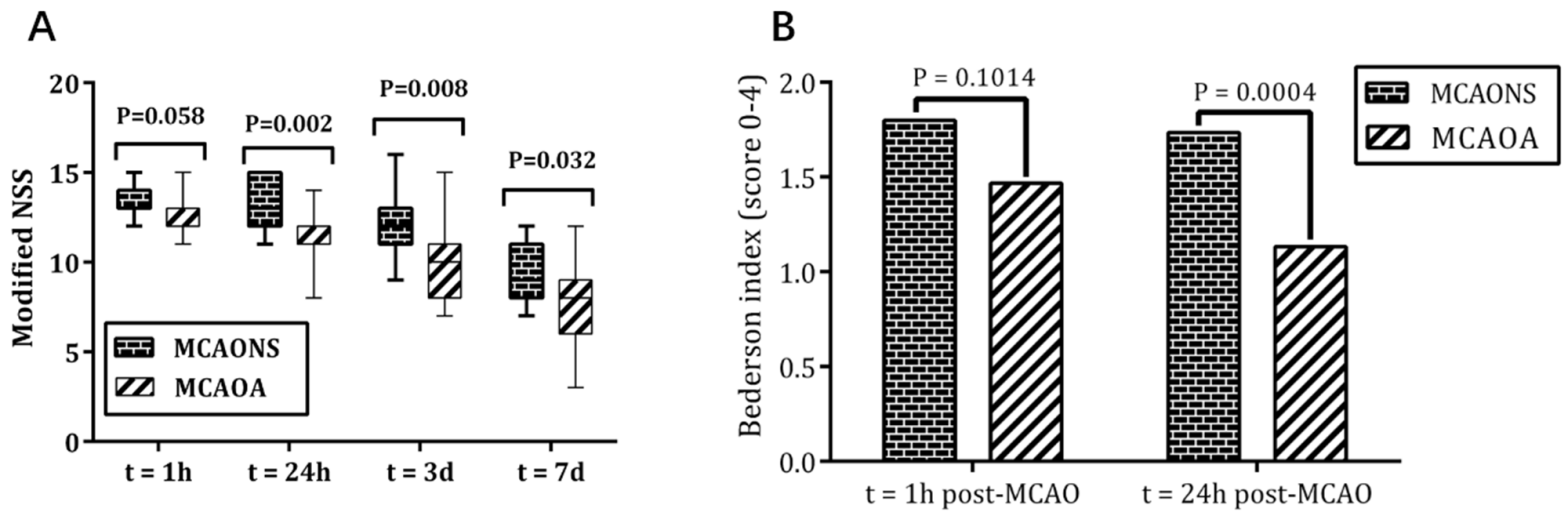
The neurological severity scores (NSS) **(A)** for MCAONS mice and MCAOA mice at 1 h, 24 h, 3 d and 7 d after MCAO, and Bederson test **(B)** for MCAONS mice and MCAOA mice at 1 h, 24 h after MCAO. ^*^P < 0.05.

Figure 2A shows the median NSS with percentiles (5% to 95%) for each group at 4 time-points. Sham-operated mice did not exhibit any neurological deficits (data not shown). In contrast, after 1 hour of MCAO, both MCAONS and MCAOA mice exhibited marked coordination dysfunction, whose median NSS scores were 14 and 13 respectively. Although the MCAOA mice had a trend toward better neurological functions (lower median NSS), the difference was not significant (MCAONS 13.60 ± 1.02 vs. MCAOA 12.73 ± 1.28; ^*^p = 0.058 > 0.05; n = 15). In the following course, significant functional improvement was observed in MCAOA group. The NSS decreased to a median score of 13 in MCAONS group and 11 in MCAOA group after 24 hours of reperfusion (MCAONS 13.40 ± 1.31 vs. MCAOA 11.60 ± 1.54; ^*^p = 0.002 < 0.05; n = 15), and gradually decreased to 9 and 7 respectively at 7 days eventually (3 days: MCAONS 12.00 ± 1.93 vs. MCAOA 9.93 ± 1.91; ^*^p = 0.008 < 0.05; 7 days: MCAONS 9.13 ± 1.63 vs. MCAOA 7.40 ± 2.36; ^*^p = 0.032 < 0.05; n = 15), suggesting that Allicin pretreatment had neurotrophic effects on IRBI.

### Bederson-based four-point scale test

After 1 h of reperfusion, both MCAONS and MCAOA mice exhibited marked coordination dysfunctions, manifesting as decreased activity, imbalance of movement, and decreased gripping ability (MCAONS: 1.80 ± 0.54 vs. MCAOA: 1.73 ± 0.44; P = 0.1014; n = 15; Figure 2B). After 24 h of reperfusion, MCAOA mice showed an improved neurological function compared with MCAONS mice (MCAONS: 1.47 ± 0.49 vs. MCAOA: 1.13 ± 0.34; ^*^P = 0.0004; n = 15; Figure 2B). Moreover, neurological deficit score of MCAOA was improved at 24 h compared with 1 h of reperfusion (24 h: 1.13 ± 0.34 vs. 1 h: 1.73 ± 0.44; ^*^P = 0.0448 < 0.05; n = 15; Figure 2B). Such improvement was not observed in MCAONS mice (24 h: 1.47 ± 0.49 vs. 1 h: 1.80 ± 0.54; P = 0.7239; n = 15; Figure 2B), suggesting an improved recovery potential in MCAOA mice. Both SONS and SOA mice did not exhibit any neurological deficit at 1 or 24 h (data not shown).

### Comparable cerebral perfusion and systemic blood pressure in MCAONS and MCAOA mice

**Figure 3:**
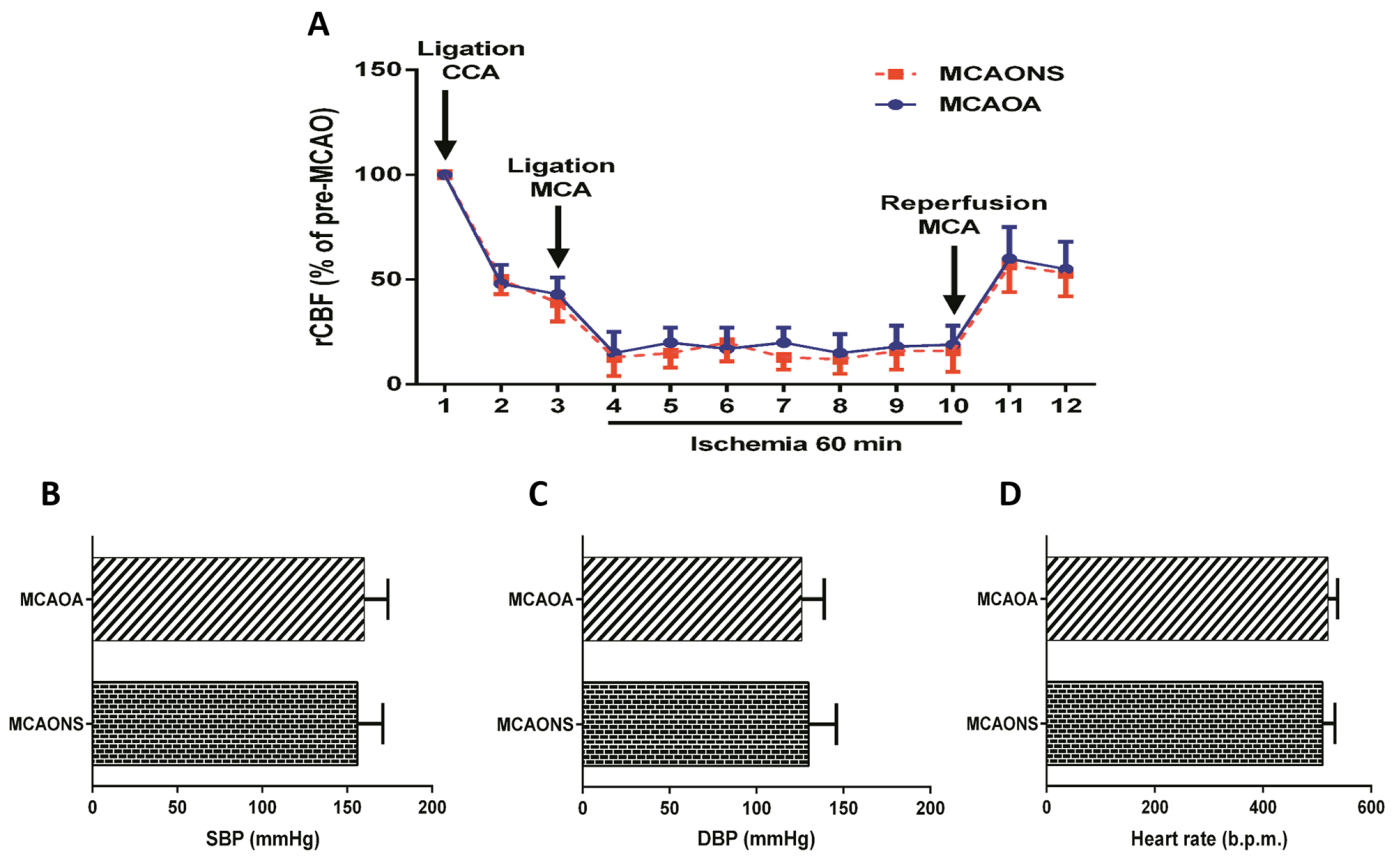
Cerebral blood flow and arterial blood pressure measurements Laser Doppler measurements revealed no difference in regional cerebral blood flow before MCAO between MCAONS and MCAOA mice and showed a similar alteration upon middle cerebral artery occlusion and reperfusion (p = NS; n = 15; **(A)**. MCAONS and MCAOA mice do not differ in systolic blood pressure (SBP; p = NS; n = 15; **(B)** and diastolic blood pressure (DBP; p = NS; n = 15; **(C)** as well as in heart rate (p = NS; n = 15; **(D)**.

Basal regional cerebral blood flow (rCBF) was comparable between MCAONS and MCAOA mice. Following the ligation of the common carotid artery (CCA), a comparable reduction in blood flow was observed in both MCAO groups (MCAONS: -52.1 ± 8.69% vs. MCAOA: -50.4 ± 7.38%; Figure 3A). Similarly, after the insertion of the silicon thread, a comparable rCBF reduction was recorded in MCAONS and MCAOA mice (MCAONS: -81.82 ± 7.49% vs. MCAOA: -85.13 ± 6.91%; Figure 3A). After the retraction of the silicone thread, a comparable re-establishment of blood flow was observed in MCAONS and MCAOA mice (MCAONS: 45.58 ± 13.12% vs. MCAOA: 47.41 ± 11.11%; p = NS for all time points; n = 15; Figure 3A). No differences in systolic blood pressure (SBP) and diastolic blood pressure (DBP) as well as in heart rate were observed in both MCAONS and MCAOA groups (SBP: MCAONS: 156.7 ± 15.67 mmHg vs. MCAOA: 160.7 ± 14.24 mmHg; p = NS; n = 15; Figure 3B; DBP: MCAONS: 131 ± 16.05 mmHg vs. MCAOA: 126.7 ± 13.21 mmHg; p = NS; n = 15; Figure 3C; Heart rate: MCAONS: 510.3+ 23.03 b.p.m. vs. MCAOA: 520.7 ± 17.88 b.p.m.; p = NS; n = 15; Figure 3D).

### Lower IRBI-induced systemic OS in MCAOA mice

**Figure 4:**
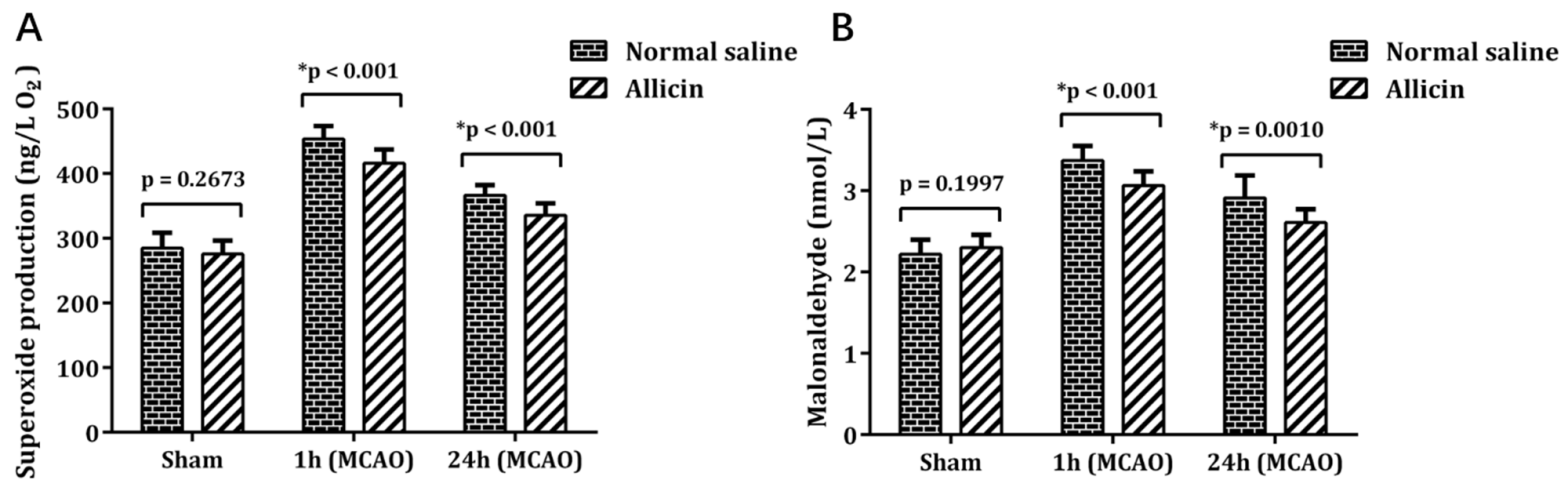
SAFR **(A)** and MDA **(B)** levels measured in whole blood using double antibody sandwich-Elisa method 1 h and 24 h after reperfusion. ^*^P < 0.05.

No significant difference of superoxide anion free radical (SAFR) and MDA were detected between SONS group and SOA group (SAFR: MCAOA 275.62 ± 20.08 vs. MCAONS 284.86 ± 23.05 ng/L; p = 0.2673; n = 15; Figure 4A; MDA: MCAOA: 2.30 ± 0.15 vs MCAONS: 2.22 ± 0.17 nmol/L; p = 0.1997; n = 15; Figure 4B). Although SAFR and MDA levels in MCAONS and MCAON groups both increased after 1 h of reperfusion, the MCAOA mice showed a lower OS degree than MCAONS mice (SAFR: MCAOA 416.15 ± 20.41 vs. MCAONS 453.66 ± 19.17 ng/L; ^*^p < 0.05; n = 15; Figure 4A; MDA: MCAOA: 3.06 ± 0.17 vs MCAONS: 3.37 ± 0.17 nmol/L; ^*^p < 0.05; n = 15; Figure 4B). After 24 h of reperfusion, SAFR and MDA followed the similar comparison trend (SAFR: MCAOA 335.48 ± 17.70 vs. MCAONS 366.41 ± 15.53 ng/L; ^*^p < 0.05; n = 15; Figure 4A; MDA: MCAOA 2.61 ± 0.16 vs. MCAONS: 2.91 ± 0.27 nmol/L; ^*^p < 0.05; n = 15; Figure 4B).

### Neuroprotection, OS, inflammation and microvessel density by immunohistochemistry

**Figure 5:**
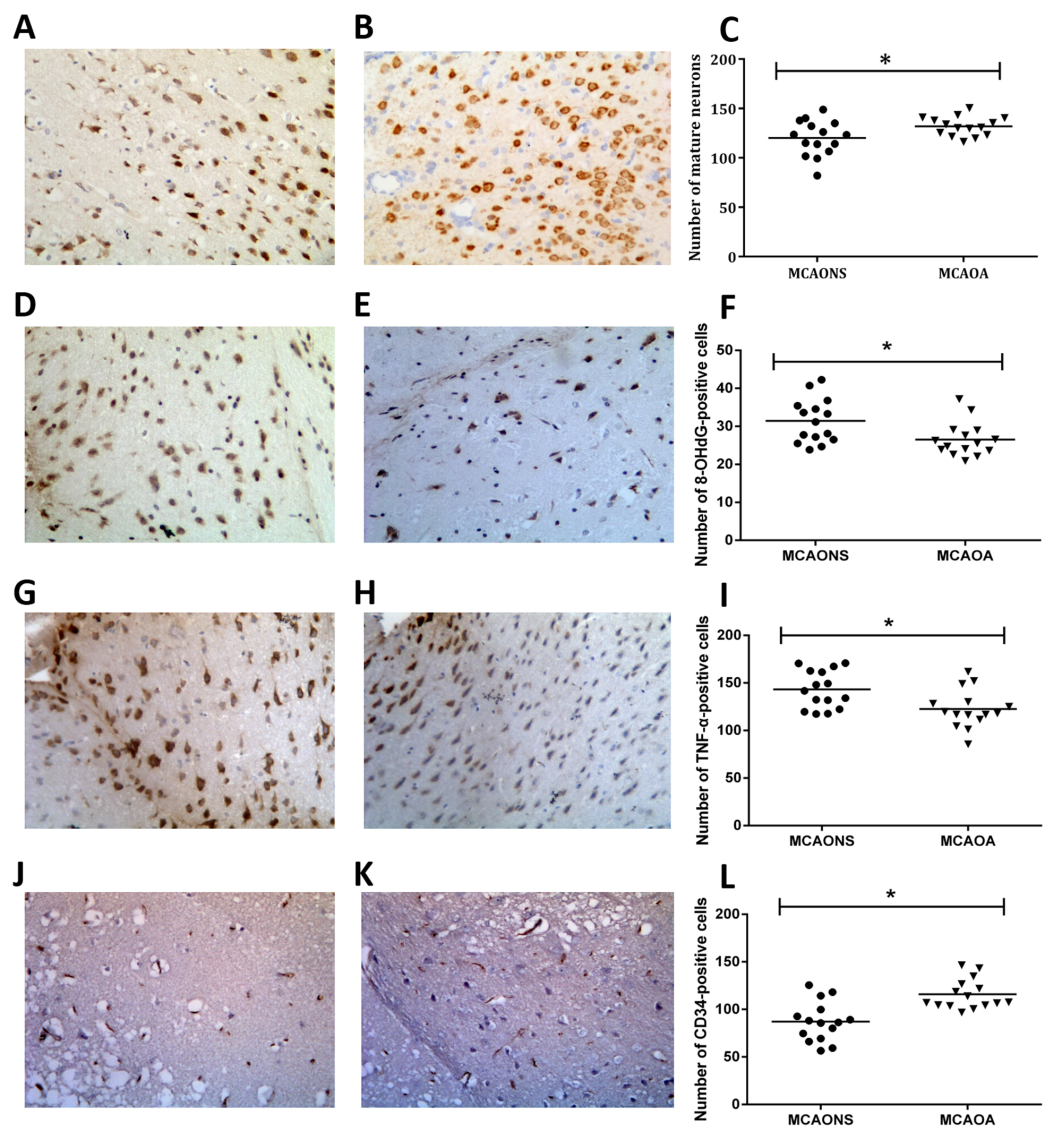
The number of mature NeuN-immunoreactive neurons was smaller in the MCAONS group **(A) t**han in MCAOA group (B) (^*^p < 0.05; n=15; **(C)** 24 h after reperfusion. There were considerably more 8-OHdG-positive cells cells in the peri-infarct zone in MCAONS **(D)** group than in MCAOA **(E)** (^*^p < 0.05; n=15; **(F)**. TNF-α followed the same pattern with 8-OHdG **(G-I)**. And the number of CD34-immunoreactive cells was smaller in the MCAONS group **(J)** than in MCAOA group **(K)** (^*^p < 0.05; n=15; **(L)**.

The number of mature NeuN-immunoreactive neurons was higher in the MCAOA group than in MCAONS group (MCAONS 120.0 ± 4.615 vs. MCAOA 132.0 ± 2.493; ^*^p = 0.0308 < 0.05; n=15; Figure 5A-5C) after 24 h of reperfusion. There were more 8-hydroxy-2’-deoxyguanosine (8-OHdG)-positive cells and TNF-α-positive cells in the peri-infarct zone in MCAONS group than in MCAOA (8-OHdG: MCAONS 31.44 ± 1.499 vs. MCAOA 26.51 ± 1.154; ^*^p = 0.0144 < 0.05; n = 15; Figure 5D-5F; TNF-α: MCAONS 143.2 ± 5.125 vs. MCAOA 122.5 ± 5.172; ^*^p = 0.0082 < 0.05; n = 15; Figure 5G-5I), suggesting the OS and inflammation could be attenuated by Allicin pretreatment. There were more CD34-positive cells in the cortical peri-infarct zones of the MCAOA group than in those of the MCAONS group after 24 h of reperfusion (MCAONS 87.17 ± 5.365 vs. MCAOA 115.9 ± 4.062; ^*^p = 0.0002 < 0.05; n= 15; Figure 5J-5L).

### Cell apoptosis assessed by immunohistochemistry

**Figure 6:**
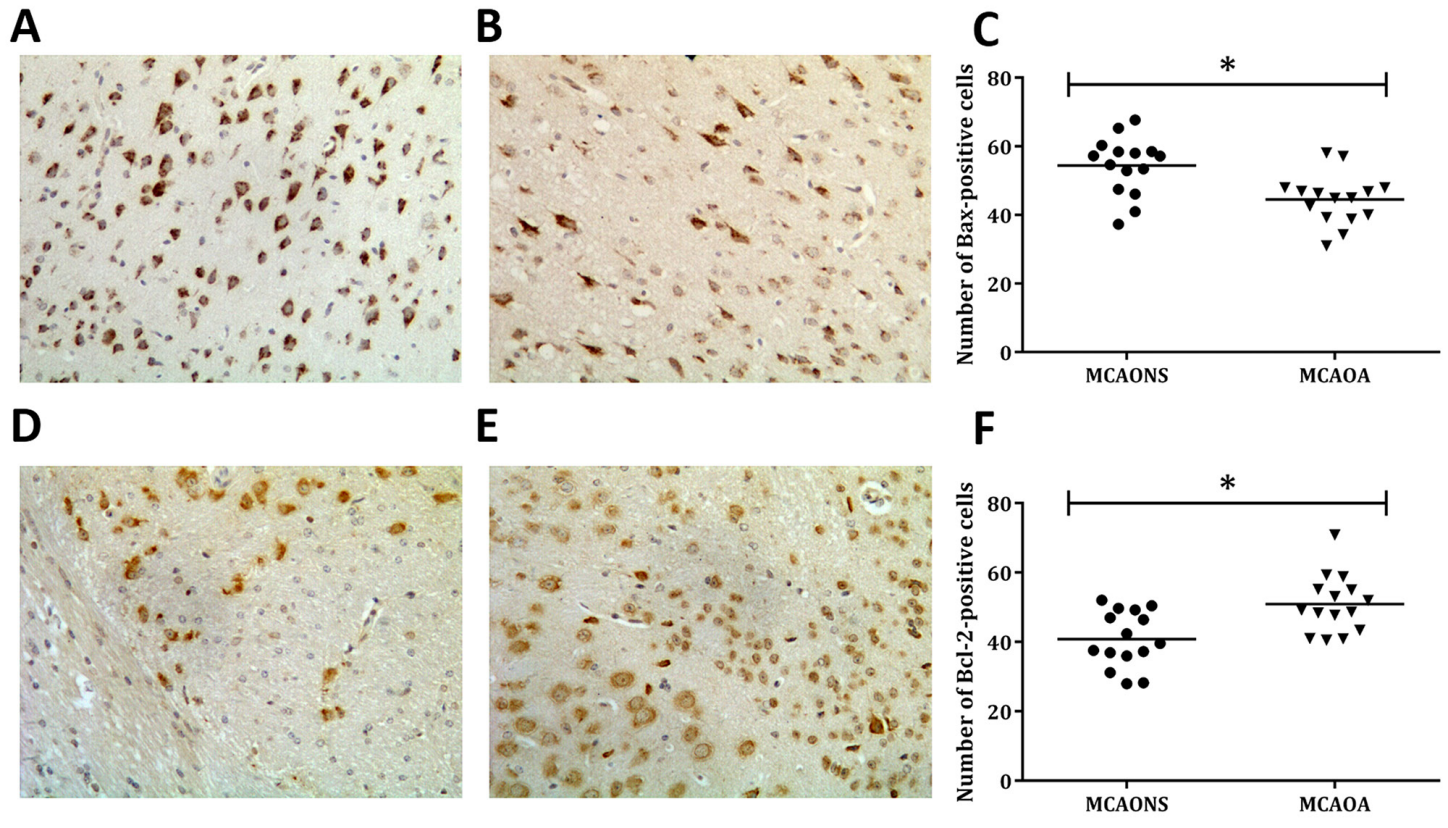
Cell apoptosis in the ischemic boundary zone shown by immunohistochemistry 24 h after MCAO Immunostaining of the proapoptotic protein Bax-positive cells in MCAONS group **(A)** and MCAOA group **(B)**. Allicin administration reduced the expression of Bax (^*^P < 0.05; n=15; **(C)**. Immunostaining of Bcl-2 positive cells in MCAONS group **(D)** and MCAOA group **(E)**. The expression of Bcl-2 in MCAOA mice was significantly higher compared to MCAONS group (^*^P < 0.05; n=15; **(F)**.

The apoptosis level and the 2 apoptosis-related proteins’ expressions were measured to confirm whether Allicin could decrease cell apoptosis. Immunohistochemical stainings for the anti-apoptotic protein Bcl-2, and the pro-apoptotic protein Bax were conducted after 24 h of reperfusion [13, 14]. The results demonstrated that the Bax expression level was much higher in the MCAONS group than in the MCAOA group (MCAONS 54.37 ± 2.166 vs. MCAOA 44.47 ± 1.891; ^*^p = 0.0018 < 0.05; n = 15; Figure 6A-6C). In contrast, the Bcl-2 expression was significantly higher in MCAOA mice than in MCAONS mice (MCAONS 40.79 ± 2.093 vs. MCAOA 50.91 ± 2.137; ^*^p = 0.0021 < 0.05; n= 15; Figure 6D-6F).

### Allicin attenuated antioxidant-enzyme consumption and mitochondrial dysfunction

**Figure 7:**
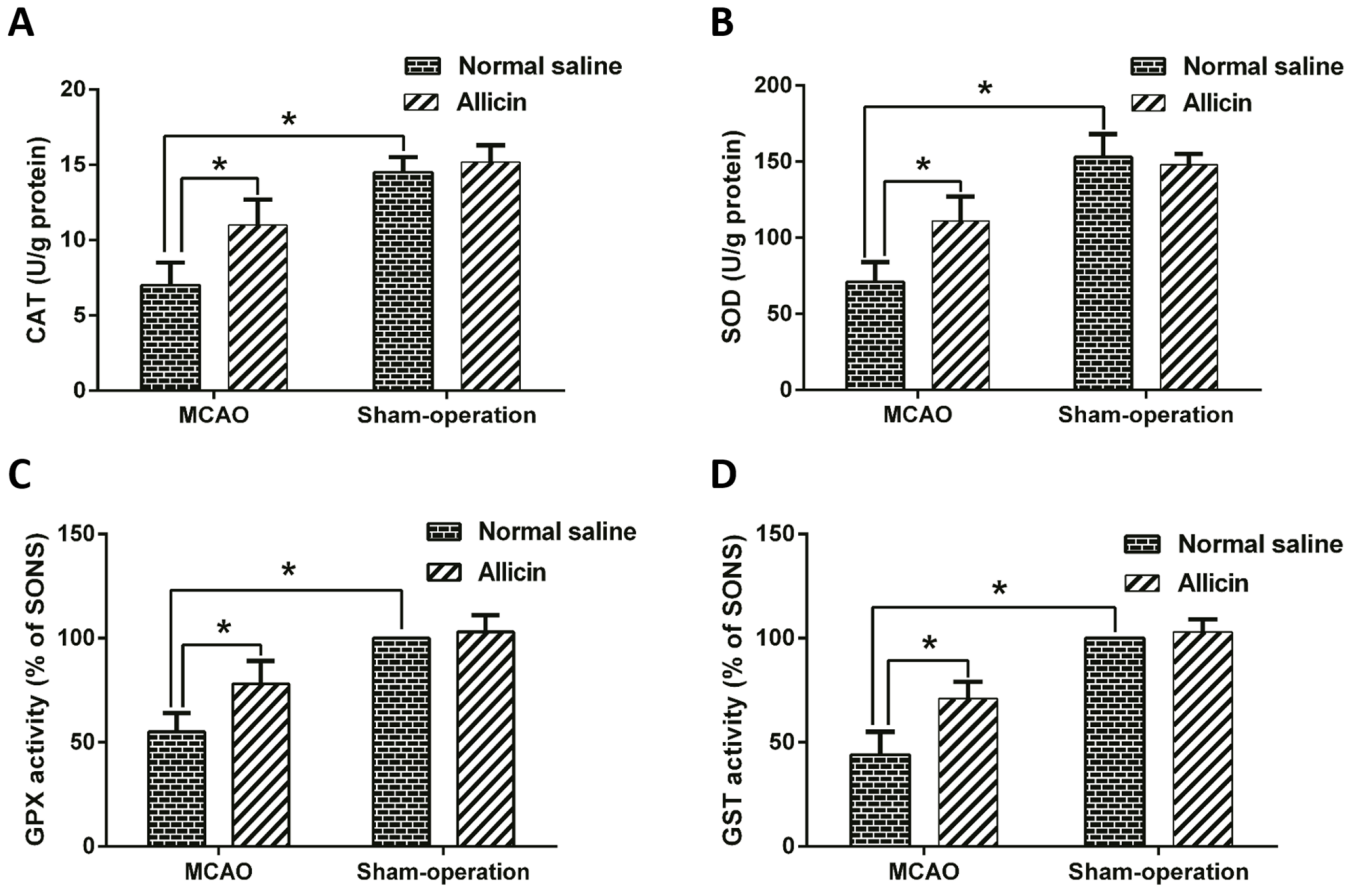
Allicin upregulated the activities of CAT, SOD, GPX and GST in MCAO injured brain After pretreatment with Allicin (50 mg/kg) for two weeks, mice were exposed to MCAO operation and sacrificed 24 h after reperfusion. The activities of CAT **(A)**, SOD **(B)**, GXP **(C)** and GST **(D)** in brain were detected. The data was represented as means ± standard error of the mean (S.E.M.). ^*^p < 0.05.

The activities of GST, GPX, SOD and CAT in ipsilateral brain homogenate were measured. The results revealed that IRBI could decrease the activities of GST, GPX, SOD and CAT markedly. Compared with MCAONS group, however, Allicin treatment (MCAOA) significantly attenuated the decrease of the activities of CAT (^*^p < 0.05; n = 15; Figure 7 A), SOD (^*^p < 0.05; n = 15; Figure 7B), GPX (^*^p < 0.05; n = 15; Figure 7C) and GST (^*^p < 0.05; n = 15; Figure 7D).

**Figure 8:**
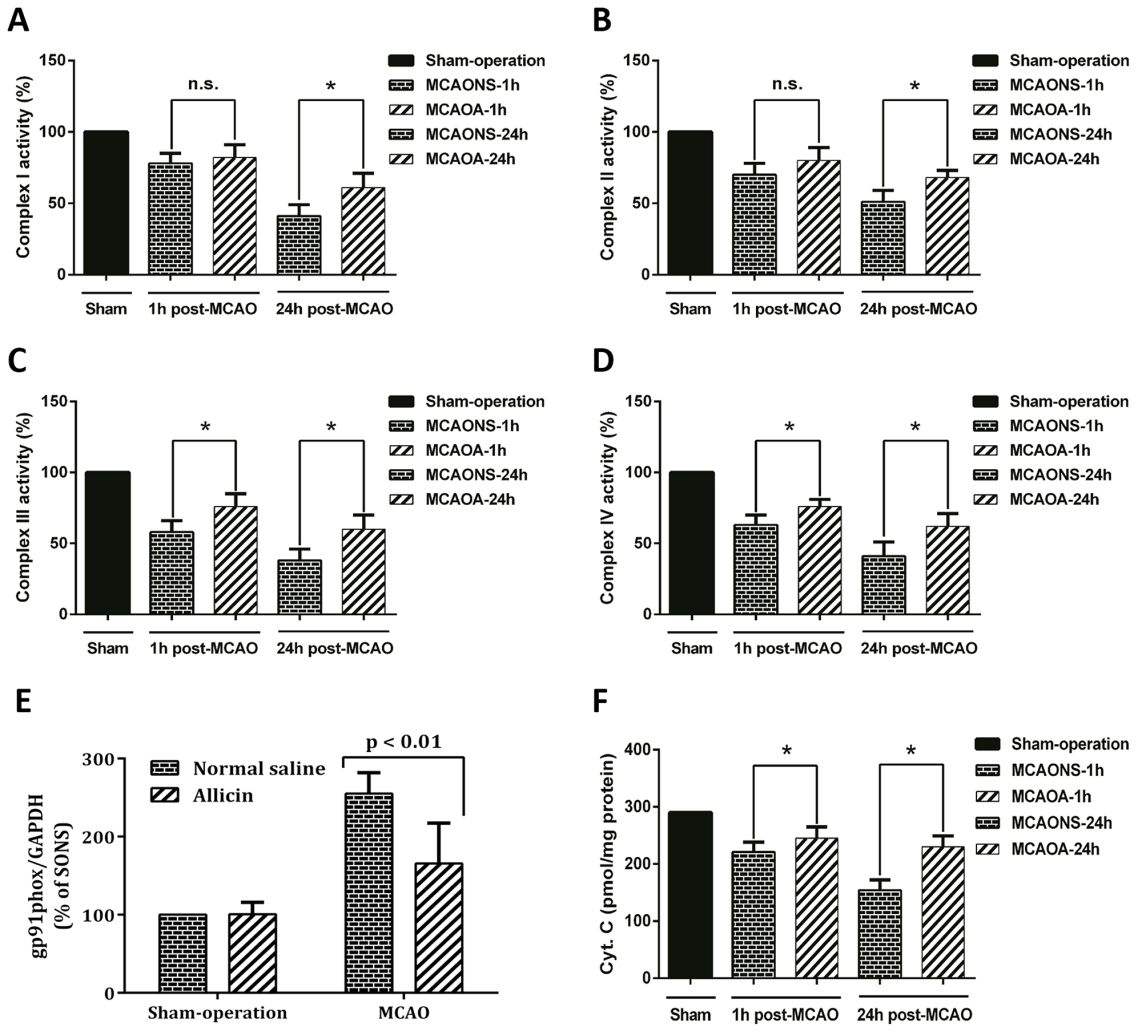
Allicin improved mitochondrial respiratory chain function in MCAOA mice brain Mitochondria were isolated from the brain tissue of MCAONS mice and MCAOA mice and the activities of mitochondrial respiratory chain complex I **(A)**, complex II **(B)**, complex III **(C)**, and complex IV **(D)** were detected at 1 and 24 h after reperfusion. Gp91phox expression was significantly increased in the brains of MCAONS mice compared with that of SONS mice **(E)**, this increase was not observed in the brain of MCAOA mice compared with that of SOA ones **(F)**. The data was represented as means ± S.E.M. ^*^p < 0.05; n.s., not statistically significant.

As compared with MCAONS mice, despite minimal effects of Allicin on the complex I and II after 1 h of reperfusion (p = NS, n = 15; Figure 8A-8B), complex III and IV showed significant increment in their activities in brain tissues from MCAOA mice (^*^p < 0.05; n = 15; Figure 8C-8D). After 24 h of reperfusion, MCAOA mice showed a remarkable augmentation of the mitochondrial electron transport chain (ETC) activities on all the four complexes (^*^p < 0.05; n = 15; Figure 8A-8D). We also measured the production of mitochondrial cytochrome c release to determine the effect of preserved ETC activity on SAFR release. Mitochondrial cytochrome c production showed significant increment in its activities in injured brain from Allicin pretreated mice (^*^p < 0.05; n = 15; Figure 8F).

### Lower gp91phox and cytochrome c in MCAOA mice

Gp91phox expression was significantly increased in the brains of MCAONS mice compared with that of SONS mice (MCAONS: 255.18 ± 25.88% vs. SONS: 100%; ^*^P < 0.05; n = 15; Figure 8E). Interestingly, this increase was not observed in the brain of MCAOA mice compared with that of SOA ones (MCAOA: 165.64 ± 50.10% vs. SOA: 100.63 ± 14.63%; P = 0.873 > 0.05; n = 15; Figure 8E). In agreement with suppression of reactive oxygen species (ROS) production and the activity of gp91phox, Allicin significantly inhibited the release of mitochondrial cytochrome c (^*^P < 0.05; n = 15; Figure 8F).

## DISCUSSION

**Figure 9:**
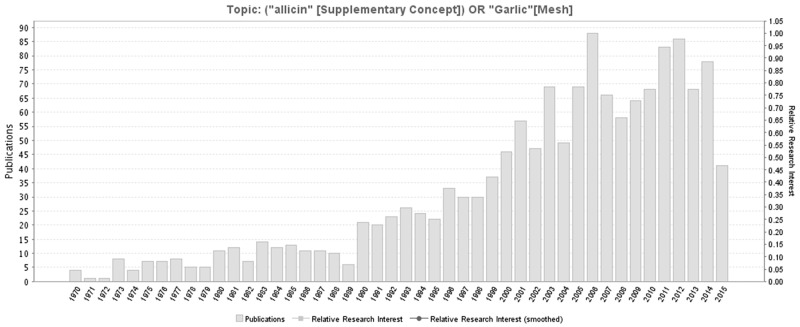
A timeline of the publications related to Allicin

**Figure 10:**
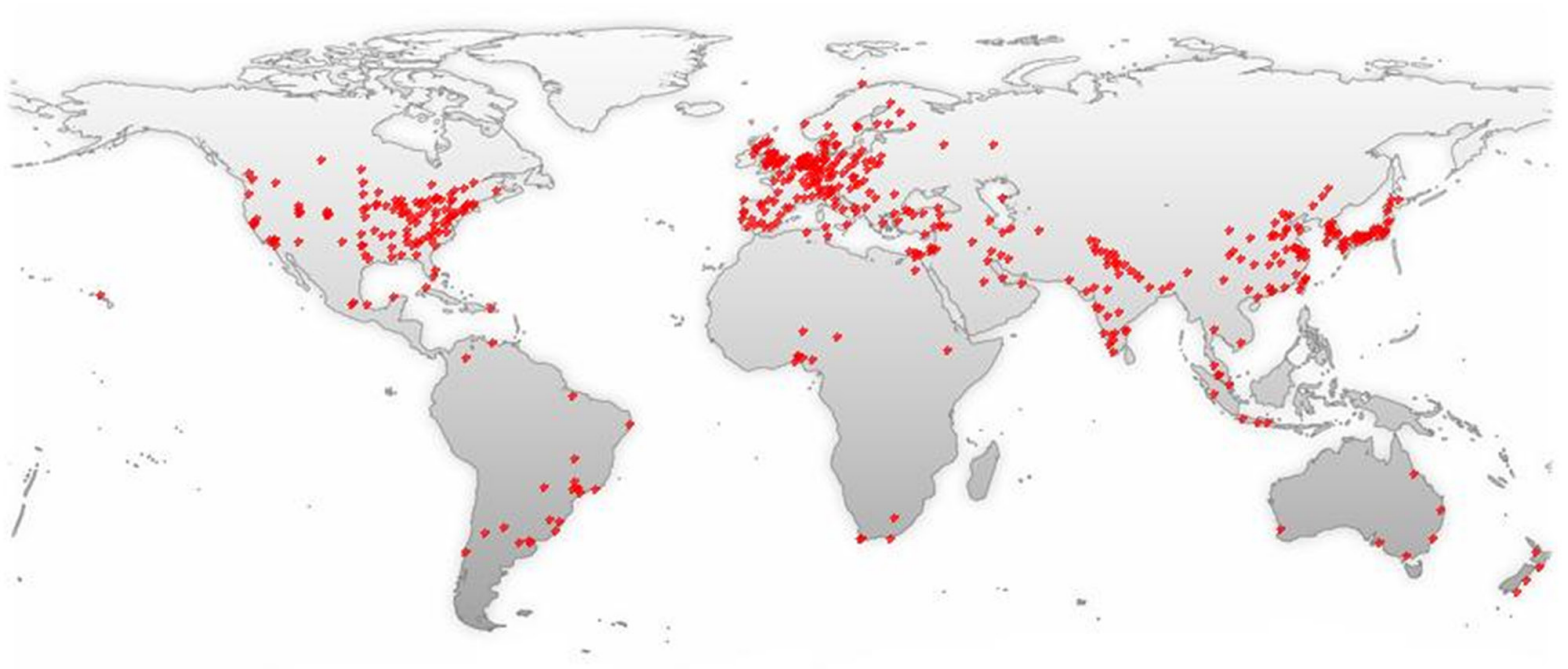
A world map with the global distribution of Allicin -related publications based on the analysis of their geolocational data

Allicin, as one of garlic’s active ingredients, possesses many medicinal functions, including anti-oxidant and anti-inflammatory activities. The number of literatures related to garlic or Allicin increases with years (Figure 9), most of which are from North America, Europe, East Asia and South Asia (Figure 10). Studies have shown that Allicin could protect against myocardial fibrosis, myocardial hypertrophy [15], and memory and learning impairments [16] through inhibiting OS-dependent signaling pathways, increasing SOD activities and reducing MDA levels. Using spinal cord ischemia reperfusion challenged rabbits, Zhu et al. found that 2 weeks’ 50 mg/kg Allicin pre-treatment reduced the infarction volumes significantly, increased motor-neuron number, and improved neurologic functions [10].

Though lots of medications with anti-oxidant activities have been shown to be able to protect against ischemia–reperfusion injury, Allicin has unique advantages. From a 50-g garlic, nearly 100 mg Allicin can be yielded [17]. Even in energy-deficient environments, Allicin, as a kind of small lipophilic molecule, can easily cross the blood-brain barrier to immediately interact with other biologically active compounds to form many beneficial metabolites [18]. In 2015, Zhang et al. published a study in Mol Med Rep that explored the protective effects of Allicin against ischemic stroke in a rat model of MCAO [11]. Rats were randomly assigned to the MCAO group, the allicin + MCAO group, and the sham-operation group. Their results demonstrated that Allicin decreased the infarction area, cerebral water content, neuron apoptosis, tumor necrosis factor (TNF-α) expression, and the activities of serum myeloperoxidase (MPO). However, the intervention in their study was administering Allicin after the MCAO procedure (at a dose of 50 mg/kg i.p. 3 h after reperfusion daily for 5 consecutive days), which could not evaluate the preventive effects of Allicin on IRBI. Secondly, since the direct effects of Allicin was anti-OS theoretically instead of anti-inflammation, only exploring the serum TNF-α level and MPO activity seems insufficient to elucidate the specific mechanism in Allicin’s protective effects. In the present study, we thus more deeply analyzed the preventative and therapeutic effects of Allicin on IRBI. This effect is paralleled by a reduced production of free radicals and inflammatory factors, an attenuated apoptosis and NADPH oxidase production, increased activities of anti-oxidant enzymes, including GST, GPX, SOD and CAT, preserved functions of mitochondria respiratory chain complexes, and attenuated ROS production and mitochondrial cytochrome c release.

Transient MCAO is a well-established model of stroke [19]. In the present study, this model induced sizeable strokes and neurological deficits. Encouragingly, Allicin administration protected mice from ischemia-reperfusion-induced brain injury. After 1 h and 24 h of reperfusion, MCAOA displayed an approximately 50% reduction of stroke volume in brain MRI examination compared with MCAONS mice. The results of immunostaining of NeuN showed there were more neurons in MCAOA mice than in MCAONS mice, suggesting that more cells survived in the peri-infarct zones of the mice pretreated with Allicin. These results expand a previously reported study that showed Allicin administration could protect against ischemia-reperfusion injured spinal cord to brain. Valuation of the neurological impairment after 1 h of reperfusion displayed a similar degree in both MCAOA and MCAONS mice. Nevertheless, after 24 h of reperfusion, MCAOA mice displayed better neurological functions than MCAONS mice. Similarly, using Bederson test, assessment of neurological deficit 1 h after MCAO denoted a similar degree of impairment in both MCAONS and MCAOA mice. However, following 24 h of reperfusion, MCAOA mice displayed a marked improvement in neuromotor function compared with MCAONS mice. This indicates that the protective effects of allicin lead to a better neurological recovery after brain reperfusion. Haemodynamic values including rCBF, heart rate and blood pressure were comparable in MCAOA and MCAONS mice, thereby excluding the impact of possible different injury degrees. Our findings have important clinical significance of translational medicine, because IRBIs are very common complications in ischemic-stroke patients receiving thrombolysis treatments [20].

Increased OS level is widely considered as a key mediator of IRBI-induced brain injury [2]. 8-OHdG is a recognized marker of DNA OS damage [21], and MDA is a biomarker for lipid peroxidation [22, 23]. Herein, we report that MCAONS mice displayed an increased SAFR and MDA production in brain tissues compared with SONS mice, demonstrating that ischemia-reperfusion injury indeed results in OS. Interestingly, brain tissue of MCAOA mice displayed much lower levels of SAFR, 8-OHdG and MDA production compared with MCAONS mice, suggesting that Allicin administration is importantly involved in the pathophysiological process. Considering the important role played by OS in IRBI, strategies aimed at preventing OS increase in this process are being sought for in recent years [24, 25]. Following ischemia, increased SAFR production promotes endothelial activations and increases brain arteries’ permeability, causing the expressions of pro-inflammatory cytokines such as TNF-α, which is an important mechanism involved in stroke size [25, 26]. During inflammation, Inflammatory cells and cytokines play important roles in the process of inflammation. Inflammation-associated cytokines include anti-inflammatory and pro-inflammatory cytokines depending on their abilities to repress or increase inflammations. Key pro-inflammatory cytokines such as TNF-α play pivotal roles in the initiation of inflammatory-reactions and leading to expressions of other cytokines after IRBI. Our results demonstrated that Allicin significantly decreased TNF-α expressions, suggesting that Allicin could protect the brain from IRBI through certain anti-inflammation pathways. Additionally, we also found that levels of SAFR and MDA in peripheral blood of MCAONS mice are much higher than those of MCAOA mice, indicating systemic ROS pathway activations under these conditions.

CD34 is a recognized biomarker for the density of micro-vessels [27]. The results of immunostaining of CD34 showed there to be more CD-34-positive cells and capillaries in the periinfarct zone in MCAOA mice than in MCAONS mice, suggesting Allicin could improve the blood perfusion by inducing neoangiogenesis and promoting the survival endothelial cells. We also explored the apoptosis degree in peri-infarct zones via bax, and bcl-2 staining. We hypothesize that Allicin’s anti-apoptosis effects are associated with its down-regulating pro-apoptotic proteins (Bax) and up-regulating anti-apoptotic proteins (Bcl-2).

Endogenous anti-oxidant enzymes are closely involved in many OS-related disorders like motor neuron disease and stroke [28]. GST, GPX, SOD and CAT can act together to form an OS-defense network. GST can reduce lipid hydroperoxides and free hydrogen peroxides to water, GPX functions as a selenium-dependent GPX, SOD can catalyze dismutations of superoxide to hydrogen peroxides, and CAT can catalyze the transformation of H_2_O_2_ to water [29]. According to our findings, Allicin pretreatment markedly imporved the activities of GST, GPX, SOD, and CAT in MCAOA mice brain, reflecting the roles Allicin played in enhancing activities of anti-oxidant enzymes. These enzymes protect against IRBI via limiting OS-mediated injuries, as well as forming a second defense line by activating down-stream metabolizing enzymes, like NQQ1 and HO-1 [29]. These data fully revealed Allicin’s vital function in upregulating anti-oxidant enzymes’ activities.

Mitochondria plays pivotal roles in producing adenosine triphosphate (ATP) and regulating cellular metabolisms via mitochondrial respiratory chain complexes. Thus, energy failure induced by mitochondria dysfunction is a significant factor in various disorders, including IRBI [30]. Studies showed that during OS conditions, the activities of complex I-IV of mitochondria decreased significantly [30, 31]. In our study, Allicin administration protected the functions of mitochondria respiratory chain complexes following MCAO. Further, Allicin attenuated the release of mitochondrial cytochrome c. The increase of cytochrome c is a major detrimental factor that activates multiple downstream signaling pathways in ischemic conditions to execute cell death.

NADPH oxidase is a membrane-bound enzyme. It is widely expressed in cerebral arteries and considered as an important source of ROS productions in cardia-cerebrovascular diseases [32]. NADPH oxidase expressions are known to increase in disease conditions such as ischemia [33], and its genetic knockout in mice could reduce brain infarction [34]. In line with these previous findings, although the expressions of gp91phox increased after reperfusion in MCAONS mice brains, the increase was not observed in MCAOA mice, suggesting that Allicin can suppress gp91phox. However, the exact mechanisms underlying the inhibiting effects of Allicin on NADPH oxidase and the involved pathways deserve further investigations.

In this study, the results of brain MRI, neurological deficit assessments, immunohistochemical examinations, western blot and SAFR and MDA tests were consistent with each other. The mechanisms by which Allicin pre-treatment prevented neuron loss might relate to the improvement of blood flow and angiogenesis, the reduction of OS and inflammation, and the repression of apoptosis. All these illustrated the preventative effects of Allicin pre-treatment.

Some limitations exist in our study. Firstly, since Zhang et al. have already explored the effects of Allicin as a post-treatment, we did not totally repeat their intervention methods, but used Allicin as a preventative measure, thus the comparison of the therapeutic effects between preoperative Allicin use and postoperative Allicin use was not performed. Secondly, there are variable neuronal functional recoveries in human patients with ischemic stroke, whether such a standard Allicin administration could also function in human patients needs further research. Thirdly, Allicin, as a molecule predominantly responsible for the antibiotic functions of garlic, exhibits a variety of pharmacological activities and is beneficial in the treatment of many disorders. Whether there are secondary effects of Allicin on humans remains unclear. Fourthly, in this study, we showed that Allicin administration protects mice from IRBI via a series of molecular biological mechanisms. Nevertheless, most of the effects of Allicin were “attenuating” but not “restoring” (except enzymatic parameters) on the morphometric, histochemical, and biochemical parameters analyzed and compared in MCAONS and MCAOA groups, so more mechanism researches on Allicin are necessary in the future. Fifthly, we only studied the effects of Allicin at the dose 50 mg/kg rather than set a dosage gradient. This is because Zhu et al. has already proved that in Allicin of 50 mg/kg has better neuroprotective effects on ischemia–reperfusion spinal cord injury than 10 mg/kg and 1 mg/kg; thus, we directly adopted the maximum dose.

## MATERIALS AND METHODS

### Animal preparation

Experiments were performed on 13–15-week-old C57 male mice. Under a dark/light cycle of 12 hours (h), experimental mice were kept at 24 °C and were fed on water and normal mouse food. Research protocols were approved by the Ethical Committee of Peking Union Medical College Hospital.

### MCAO model and hemodynamics monitoring

As previously described, a transient MCAO procedure was conducted to induce IRBI on experimental mice. Sham-operated mice underwent the same procedures except without interruption of cerebral blood flow (CBF) in the middle cerebral artery (MCA) [19, 35]. During operation, using laser Doppler flowmetry, we measured the regional CBF (rCBF) in cortex supplied by MCA. To exclude possible interference on stroke volume by the differences of systemic blood pressure, heart rates and blood pressure were measured according to procedures described by Spescha et al [24]. The experiment was performed blindly.

### Experimental grouping design

Mice were assigned to 4 groups: (a) sham-operated Allicin (SOA) group: pretreated with Allicin 50 mg/kg by intraperitoneal injection per day for 2 weeks. The same procedures with MACO model making were performed for sham-operated animals. However, the silicone-coated filament was advanced into the internal carotid artery for 5 mm from the common carotid bifurcation, without interruption of cerebral blood flow in the middle cerebral artery; (b) sham-operated normal saline (SONS) group: the difference between SOA group and SONS group is that mice in SONS group were pretreated with normal saline of the same volume; (c) MCAO Allicin (MCAOA) group: pretreated with Allicin 50 mg/kg by intraperitoneal injection per day, for 2 weeks, and underwent MCAO procedures; (d) MCAO normal saline (MCAONS) group: pretreated with normal saline of the same volume with MCAOA group, and underwent MCAO procedures. The neuroprotective effects of Allicin were assessed using behavioral and histological techniques as described below. Allicin (purity >98%) was purchased from the National Institute for the Control of Pharmaceutical and Biological Products (Beijing, China). Allicin was dissolved in 2% dimethyl sulfoxide (DMSO; Sigma-Aldrich). There were 75 mice in each group. Among the 75 mice, 15 mice were for neurological deficit measurement (1h, 24h, 1d, and 7d) and the measurements of superoxide anion free radical (SAFR) and malondialdehyde (MDA) in blood (1h and 24h); 15 mice were for MRI scanning and immunohistochemical examinations (24h); 15 mice were for the measurements of anti-oxidant enzymes, NADPH subunit gp91phox, and cytochrome c; and the remaining 30 mice were for the measurements of mitochondrial respiratory chain complexes activities (1h and 24h, 15 mice respectively).

### Neurological deficit measurements

#### NSS

The neurological severity score (NSS, its brief descriptionis presented as Table 1) evaluation was performed 1 hour, 24 hour, 3 days and 7 days after MCAO blindly [36]. Neural functions were graded as 18 to 0 (maximal deficit score: graded as 18; normal score: graded as 0). Neurological deficit measurements were performed by 2 investigators independently and blindly. Number of samples for each group: 15.

**Table 1:** Neurological Severity Scores (NSS)

Evaluation Items	Points
**Motor tests**	-
**Raising rat by the tail**	3
1 Flexion of forelimb	-
1 Flexion of hindlimb	-
1 Head moved >10° to vertical axis within 30 s	
**Placing rat on the floor (normal=0; maximum=3)**	3
0 Normal walk	-
1 Inability to walk straight	-
2 Circling toward the paretic side	-
3 Fall down to the paretic side	-
**Sensory tests**	2
1 Placing test (visual and tactile test)	-
2 Proprioceptive test (deep sensation, pushing the paw against the table edge to stimulate limb muscles)	
**Beam balance tests (normal=0; maximum=6)**	6
0 Balances with steady posture	-
1 Grasps side of beam	-
2 Hugs the beam and one limb falls down from the beam	-
3 Hugs the beam and two limbs fall down from the beam, or spins on beam (>60 s)	-
4 Attempts to balance on the beam but falls off (>40 s)	-
5 Attempts to balance on the beam but falls off (>20 s)	-
6 Falls off: No attempt to balance or hang on to the beam (<20 s)	-
**Reflexes absent and abnormal movements**	4
1 Pinna reflex (head shake when touching the auditory meatus)	-
1 Corneal reflex (eye blink when lightly touching the cornea with cotton)	-
1 Startle reflex (motor response to a brief noise from snapping a clipboard paper)	-
1 Seizures, myoclonus, myodystony	
**Maximum points**	18

One point is awarded for the inability to perform the tasks or for the lack of a tested reflex; 13 to 18 indicates severe injury; 7 to 12, moderate injury; 1 to 6, mild injury.

#### Bederson test

Using a 4-point scale according to the Bederson test, neurological deficit measurements were also performed at 1h and 24 h after reperfusion as a supplement. The scoring criteria was based on what Bederson described [37]. This test was also conducted by 2 investigators independently in a blinded way. Number of samples for each group: 15.

### Measurement of reactive oxygen species and malondialdehyde in blood

Superoxide anion free radical (SAFR) level can directly reflect the OS degree [38]. Malondialdehyde (MDA) is a bio-marker for lipid-peroxidation [22, 23]. SAFR and MDA production in whole blood was tested using the double antibody Sandwich-Elisa method after 1 h and 24 h of reperfusion. Blood was sampled from mouse eyes. We firstly used purified mouse SAFR antibody and MDA antibody to coat microtiter plate wells to make solid-phase antibody, then added SAFR and MDA to wells, respectively. Then the tetramethylbenzidine substrate was added which could become blue under the catalysis of mouse radish peroxidase. Reactions were terminated by adding the sulphuric acid solution which could promote the color to change to yellow. Within a certain extent, the color depth is correlated with the SAFR and MDA content. The optical density (OD) was measured spectrophotometrically at a wave-length of 450 nm. The concentrations of SAFR and MDA in blood were determined by comparing the OD of the samples to the standard curve. Number of samples for each group: 15.

### Infarct volume measurement

After 24 h of reperfusion, animals were anesthetized using 10% chloral hydrate and underwent a brain MRI scanning (T2-weighed image, T2WI) by a 3T animal MRI-scanner (Bruker BioSpin MRI PharmaScan) [12, 39]. The mice would be prostrated on a custom-made holder with strapping to minimize head motions. Coronal MRI sections were conducted from 2mm anterior to the corpus callosum to the cerebrum end. Parameters: field of view (FOV) = 2.5 × 2.5 cm^2^, echo time (TE) = 20 ms, repetition time (TR) = 11189 ms, slice thickness = 1.0 mm, and matrix size = 128 × 128. Volumes of intact zones were measured in cubic millimeters by summing areas and multiplying by the distance between sections blindly. Number of samples for each group: 15.

### Immunohistochemical assessment

Twenty-four hours after MCAO, mice were anesthetized using 10% chloral hydrate and perfusion-fixed using 4% paraformaldehyde in PBS (0.1 mol/L). The brains were removed rapidly, immersion-fixed in 4% paraformaldehyde for 4 hours at 4°C and cryo-protected using 30% PBS/sucrose (72h, 4°C). The brains were cut into coronal sections (thickness: 30 μm) on a freezing microtome (SM 2000R; Leica, Nussloch, Germany). Sections were incubated with 1.5% normal blocking serum for 1 h at normal temperature, and overnight at 4°C for immunohistological staining with hematoxylin and eosin and antibodies to neuronal nuclear antigen (NeuN; 1:500; Abcam), 8-hydroxy-2’-deoxyguanosine (8-OHdG; 1:400; GeneTex, San Antonio, TX, USA), Tumor necrosis factor-alpha (TNF-α; 1:500; Beyotime), CD34 (1:500; Beyotime), Bcl-2 (1:200; Sigma) and Bax (1:200; Sigma). NeuN was used to estimate the numbers of remnant mature neurons in peri-infarct zones [40]. 8-OHdG was used to investigate DNA oxidative damage [21]. TNF-α could reflect the recruitment of inflammatory cells into the brain parenchyma [41]. CD34 reflects the microvessel density and angiogenesis in the ischemic boundary zone [27]. Bax and Bcl-2 were adopted to analyze and compare the degrees of apoptosis [13]. Secondary antibodies and visualization were performed using the ImmPRESS Universal (mouse/rabbit) Ig Kit (Vector Laboratories, Burlingame, CA, USA). The sections were imaged through specific analysis systems (Leica TCSSP2, Leica, Wetzlar, Germany). Immuno-reactive cells were counted based on the evaluation of an average of 3 slides from each mouse using ImagePro Plus software (Media Cybernetics, Rockville, MD, USA). The positive cells in the peri-infarct region were counted with a 20 × objective. The areas in each slice only with the densest positive cells would be chosen for counting. Number of samples for each group: 15.

### Measurements of anti-oxidant enzymes activities

In OS process, various endogenous anti-oxidant enzymes form an anti-oxidant network to protect cells against apoptosis and necrosis [42]. Based on the instructions intechnical manuals of detection-kits (Cayman Chemical, USA), we estimated the activities of anti-oxidant enzymes including glutathione S-transferase (GST, expressed as the percentage of control), superoxide dismutase (SOD, expressed as U/mg protein), glutathione peroxidase (GPX, expressed as the percentage of control) and enzyme activities of catalase (CAT, expressed as U/g protein) in brain homogenates [42]. Number of samples for each group: 15.

### Measurements of mitochondrial respiratory chain complexes activities

Mitochondrial respiratory chain complex dysfunction has been demonstrated to be one of the most critical influences on IRBI [43]. To elucidate the correlations between Allicin’s neuroprotective effects and mitochondrial dysfunction, we firstly measured the activities of complexes I–IV of mitochondrial electron transport chain (ETC) in injured brain tissues. Brain tissues were obtained at 1 or 24 h after reperfusion, and mitochondria were purified as described earlier [44]. It would take 3 freeze-thaw cycles to disrupt membranes and have enzymes exposed. Four enzymes were selected to be measured at 37 °C (presented as the percentage of control): NADH dehydrogenase (complex I), succinate dehydrogenase (complex II), ubiquinol cytochrome c reductase (complex III), and cytochrome c oxidase (complex IV) [45]. Number of samples for each group: 30 (1h and 24h, 15 mice respectively).

### Mitochondrial NADPH production

NADPH oxidase is a recognized main source for OS in cardia-cerebrovascular diseases. Thus, NADPH oxidase expressions were measured in brain tissue homogenates after 24 h of MCAO [32]. NADH expressions were estimated through western blot. The homogenate tissues that were analyzed were isolated cerebral hemispheres. Antibodies against gp91phox, the key subunit, were used at 1:500 dilution. The operation steps and the related reagents used are the same with what Spescha et al. decribed [24]. Number of samples for each group: 15.

### Measurement of cytochrome c

Contents of mitochondrial cytochrome c were measured and analyzed according to the steps described by Zhu et al. [10]. After 24 h of MCAO, the brain tissues were supplemented with bovine serum albumin to an ultimate concentration of 25 μm. The amounts of cytochrome c were calculated based on the integrated chromatographic peak area [46]. Number of samples for each group: 15.

### Statistical analysis

Statistical analyses were performed using two-way ANOVA analysis of variance. P<0.05 was considered to indicate a significant difference. Statistical analysis and charting were performed using the GraphPad Prism software version 6 (GraphPad Software, Inc., La Jolla, CA, USA) and the IBM SPSS 19.0 software package (SPSS, Inc., Chicago, IL, USA).

## CONCLUSIONS

In summary, our findings showed that Allicin administration could significantly reduce stroke size following IRBI. In line with this, MCAOA mice showed a much milder neurological impairment compared with MCAONS. This protective effect is likely to be mediated by a reduced production of free radicals and inflammatory factors, an attenuated apoptosis, a blunted activation NADPH oxidase, and an improvement of antioxidant enzymes, mitochondrial respiratory chain and cytochrome c. As a natural product, garlic is a widespread dietary component with no reported toxicities. Allicin represents unique advantages and may be an effective and novel drug in preventing IRBI in patients with ischemic stroke undergoing thrombolysis of interventional reperfusion therapies. Future studies including larger animal models, longer follow-up as well as clinical experiments should be performed.

### Ethical approval

All applicable international, national, and/or institutional guidelines for the care and use of animals were followed.

## Abbreviations

IRBI: Ischemia-reperfusion brain injury
OS: oxidative stress
MCAO: middle cerebral artery occlusion
MRI: Magnetic Resonance Imaging
NeuN: neuronal nuclear antigen
NADPH: nicotinamide adenine dinucleotide phosphate
CAT: catalase
SOD: superoxide dismutase
GPX: glutathione peroxidase
GST: glutathione S-transferase
TNF: tumor necrosis factor
MPO: myeloperoxidase
NSS: neurological severity score
rCBF: regional cerebral blood flow
CCA: common carotid artery
SBP: systolic blood pressure
DBP: diastolic blood pressure
NS: no significance
SAFR: superoxide anion free radical
MDA: malondialdehyde
8-OHdG: 8-hydroxy-2’-deoxyguanosine
ETC: mitochondrial electron transport chain
ROS: reactive oxygen species
